# Defect-mediated n-type to p-type transition and enhanced thermoelectric performance in Bi_1.8−*x*_Ni_*x*_Sb_0.2_Te_3_ alloys

**DOI:** 10.1038/s41598-026-54090-2

**Published:** 2026-05-25

**Authors:** G. Poojitha, P. Poornesh, Ashok Rao, C. L. Hung, Y. K. Kuo, Om Prakash, Dhanya Sunil

**Affiliations:** 1 Manipal Institute of Technology, Manipal Academy of Higher Education, Manipal, 576104 India; 2 Department of Physics, National Dong-Hwa University, Hualien, 974 Taiwan; 3 Department of Physics, Sri Aurobindo College, University of Delhi, New Delhi, 110017 India

**Keywords:** Bi–Sb–Te thermoelectric alloys, Nickel doping, Power factor, Mass disorder scattering, Low-temperature thermoelectric transport, Materials science, Physics

## Abstract

A systematic investigation pertaining to the low temperature (10–350 K) thermoelectric properties of Ni substituted Bi_1.8−*x*_Ni_*x*_Sb_0.2_Te_3_ alloys (*x* = 0–0.08), it was synthesised employing solid state reaction method. X-ray diffraction studies verified the presence of phase pure rhombohedral structures. FESEM micrographs showed a morphological transition from hexagonal platelets to granular networks as the Ni content increased. Electrical transport measurements revealed a transition from n-type to p-type conduction for the sample *x* ≥ 0.06. This transition is attributed to the formation of acceptor defects, which increases the hole concentration as a majority charge carriers. The thermal conductivity decreased systematically from 1.7 to 1.0 Wm^−1^ K^−1^ at 350 K with substitution of Ni concentration, as a result of the increased phonon scattering due to the mass disorder and strain fields. The optimal composition (*x* = 0.04) unveiled a maximum *PF* and *ZT* of 335 μW/mK^2^ and 0.09 respectively at 350 K, *ZT* has 125% increment over the pristine sample. These results validates that Ni doping effectively decouples thermal and electronic transport properties via controlled defect engineering. This controlled doping represents a viable strategy for advancing thermoelectric performance of Bi–Sb–Te system.

## Introduction

Thermoelectric (TE) devices composed of TE material have a potential for the direct conversion of thermal energy into electrical energy and vice versa. This characteristic makes them a potentially effective avenue for harvesting waste heat and for utilized in solid-state cooling devices^1,2^. The TE figure of merit (*ZT*) parameter that quantifies the material efficiency and is governed by the properties such as Seebeck coefficient, electrical conductivity and thermal conductivity. The ideal TE materials should have high Seebeck coefficient, low electrical resistivity and thermal conductivity. But these transport variables are inseparably interdependent that is obstructing to enhance the *ZT* value for TE materials^3^. Hence the engineering of new materials or the optimization of presently established materials through various strategies, such as inducing defects, modifying the band structure, alloying and engineering interfaces has become a primary focus in current thermoelectric research^4^. Remarkable progress has been achieved in different class of TE materials, such as Chalcogenides, skutterudites, half-Heusler, oxides, clathrates and silicides. These have shown the significant TE performance over the past decades^5,6^. More recently, organic, polymeric and hybrid thermoelectric, together with flexible and printed architectures, have emerged as promising candidates for low‑temperature operation and wearable devices owing to their mechanical compliance and low‑cost processing^7–9^. These improvements have enabled the development of the high performance of TE devices for various applications, including industrial waste-heat recovery, automotive exhaust energy harvesting, space power generation, refrigeration, cooling in electronic and optoelectronic systems, low‑power sensor networks and wearable electronic and biomedical systems^10,11^.

Among these material systems, bismuth telluride and antimony telluride alloys (Bi_2_Te_3_–Sb_2_Te_3_) remain the standard for mid-temperature TE applications^12^. Their layered crystal structure encompasses stacked quintuple units across the *c*-axis with weak van der Waals gaps. This structure facilitates anisotropic charge and heat transport, making these alloys exemplary for high-performance modules in refrigeration and power generation devices^13–15^. Binary Bi_2_Te_3_ exhibits a narrow band gap, significant spin–orbit coupling and a tendency for defect formation which in combination influence its electronic and phononic properties^16,17^. Alloying with Sb_2_Te_3_ not only modulates the band structure and also introduces mass disorder which successively reduces lattice thermal conductivity and optimizes TE performance^18,19^.

There are reports that suggest that through the stoichiometric tuning of Bi_2-*x*_Sb_*x*_Te_3_ alloys, the n ↔ p transition can be observed based on the ratios of Bi and Sb^20–22^. Thereby, research on Bi_1.8_Sb_0.2_Te_3_ has demonstrated that the diffusion of Bi atoms and the type of conductivity can be controlled by annealing temperature. Optimizing the carrier concentration has resulted in a high-performance thermoelectric material^23^. Im et al. have shown that optimizing the TE properties of the Bi_1.8_Sb_0.2_Te_3_ alloy can enhance efficiency near room temperature (200–400 K)^24^. Ghose *et* *al*. observed a high magneto-Seebeck coefficient at room temperature for n-type conductivity via anionic disorders^25^. The stoichiometric tuning of the Te content, which induces anionic disorder in the Bi_1.8_Sb_0.2_Te_3_ alloy has been reported to increase TE efficiency in both n-type and p-type conductivity^26^. This development has sparked interest in the investigation of cationic disorders within the system. The literatures extensively documented substitutional doping strategies at the cationic site of Bi–Sb–Te alloy with predominantly targeting Sb-rich compounds^27^.

The introduction of transition metal dopants into the Sb sublattice of Bi_0.5_Sb_1.5_Te_3_ considerably elevates the TE performance by concurrently refining electronic and phononic transport mechanisms. When small amounts of Ti, Fe, Ni and Mn replace Sb creates a point defects that proficiently scatter mid-high frequency phonons contributing to a reduction in lattice thermal conductivity^28–32^. In parallel, these dopants serve as acceptor impurities by that varying the hole concentration and increasing the carrier effective mass^31,33^. These coupled mechanisms culminate in an improved Seebeck coefficient while preserving electrical conductivity at a desirable level. In the present work Ni has been selected as a dopant, as it acts as an effective barrier layer at cold-end interfaces. It further provides a superior thermal tolerance and 50% higher bonding strength compared to conventional materials by maintaining low contact resistivity^34^. The intrinsic magnetic properties of Ni^2+^ ions contribute to high TE performance through spin-entropy effects and magnon drag mechanisms that significantly boost the Seebeck coefficient^35–37^. Nickel as a dopant forms a thermodynamically stable NiTe_2_ secondary phases that induces a beneficial charge carriers for carrier filtering as well as additional phonon scattering centres^38,39^. These integrated combination of Nickel makes it a strategic dopant for comprehensive TE optimization^40^.

Recently we reported the effect of Ni composites for Bi–Sb–Te alloys where inclusions of Ni into the Bi–Sb–Te matrix degrades the thermoelectric efficiency of the pristine sample. Because of the enhanced electron scattering and a marked decrease in carrier mobility arising from metal–semiconductor interface barriers, while metallic Ni acted as an efficient heat-conduction pathway and increased the overall thermal conductivity of the system. These results underscore the inherent limitations of composite strategies involving metallic inclusions in n-type Bi–Sb–Te system. The decrease in ZT observed in the Ni composites prompted us to explore doping as an alternative strategy^41^. However, Bi–Sb–Te alloys have been profoundly investigated at ambient temperature but research into their low-temperature transport properties remains limited additionally concerning the effects of transition-metal doping on n-type compositions. Accordingly, this study systematically evaluates the consequences of Ni substitution mechanism on the structural, microstructural and low-temperature TE properties of Bi_1.8-*x*_Ni_*x*_Sb_0.2_Te_3_ (0 ≤ *x* ≤ 0.08). And noted a distinct conversion from n-type to p-type conductivity with an elevation of the Seebeck coefficient. Along with the significant mass disorder scattering effect contributes to a suppression in thermal conductivity accompanied by an enhancement of the figure of merit.

## Methodology

**Fig. 1:**
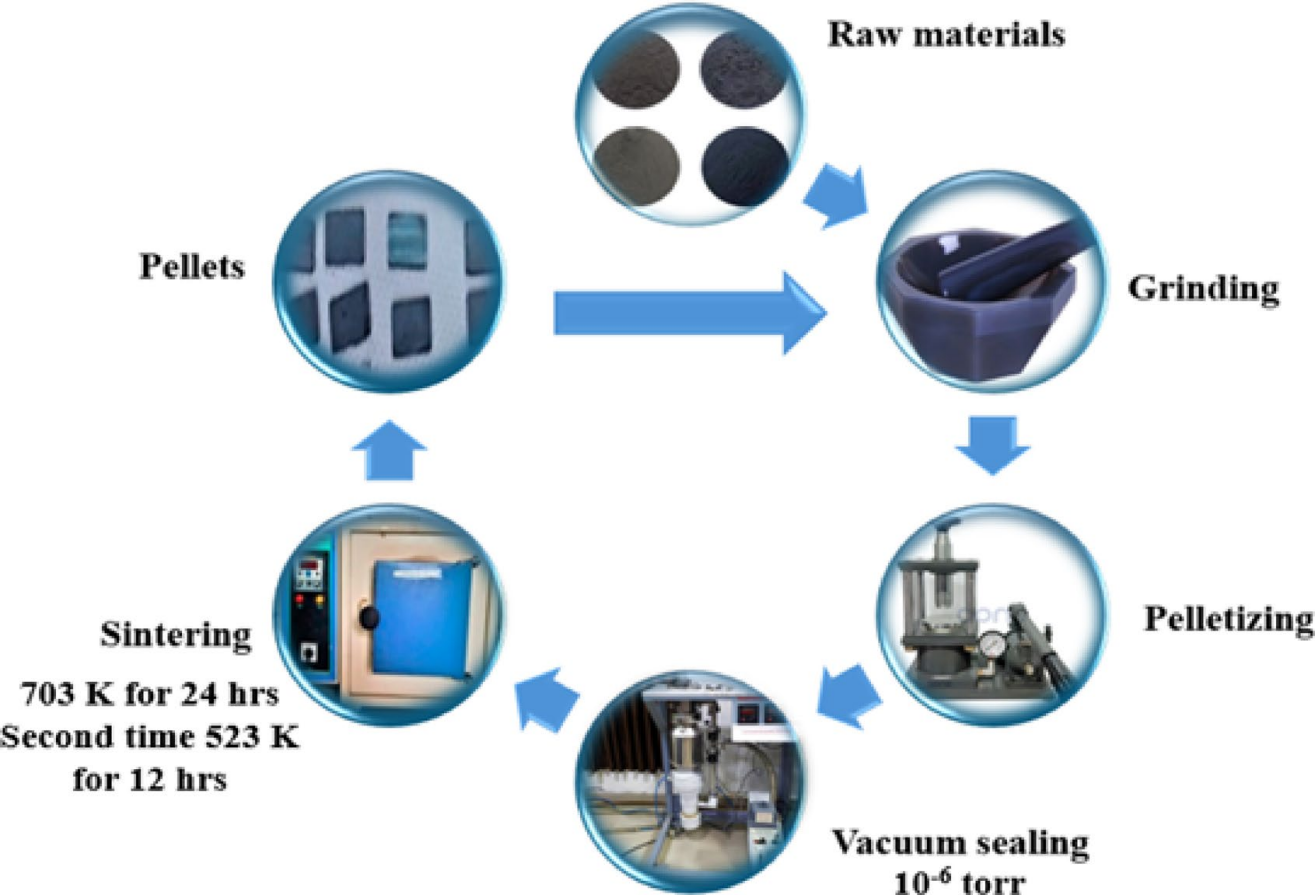
Experimental workflow of bulk Bi_1.8-*x*_Ni_*x*_Sb_0.2_Te_3_ samples.

The polycrystalline Bi_1.8-*x*_Ni_*x*_Sb_0.2_Te_3_ (*x* = 0, 0.02, 0.04, 0.06, 0.08) samples were prepared via a two-step solid-state approach. The required high purity raw materials are Bismuth (Bi) (99.5% Alfa aesar), Antimony (Sb) (99.999% Good fellow), Nickel (Ni) (99.9%, Alfa aesar) and Tellurium (Te) (99.999% Alfa aesar). The description of experimental procedure is schematically demonstrated in the Fig. 1.

The structural and TE properties of the synthesised samples were characterized using a comprehensive suite of analytical techniques. Crystallographic analysis carried out utilizing a Rigaku Miniflex X-ray diffractometer to determine structural phase purity and crystallinity. Morphological features were examined via Field emission scanning electron microscopy (FESEM) using Thermofisher Apreo 2 s system operating in Optiplan mode with the simultaneous elemental composition determined through Oxford instrument energy dispersive X-ray spectroscopy (EDX). The photoemission spectra were acquired on a Thermo Scientific XPS instrument using monochromatic Al Kα with the 200 eV pass energy and the range of 0–1300 eV Binding energy. Casa XPS software was used for peak deconvolution using Shirley type background. Transport properties were probed using a four-probe resistivity measurement system across the temperature 10–350 K with the standard error of ± 3%, concurrently acquiring Seebeck coefficient and thermal conductivity measurement via direct pulse heating technique with the experimental standard error of ± 5%. Room temperature Hall measurements were conducted with an Ecopia HMS-5500 apparatus with a 0.5 T magnetic field to measured carrier concentration and mobility. Density measurements were determined by the Archimedes method using the DI water as an immersion liquid and possible estimated error is ± 2%. The porosity was estimated by using the ratio of measured bulk density and the theoretical density of the samples.

## Results and discussion

### XRD

**Fig. 2:**
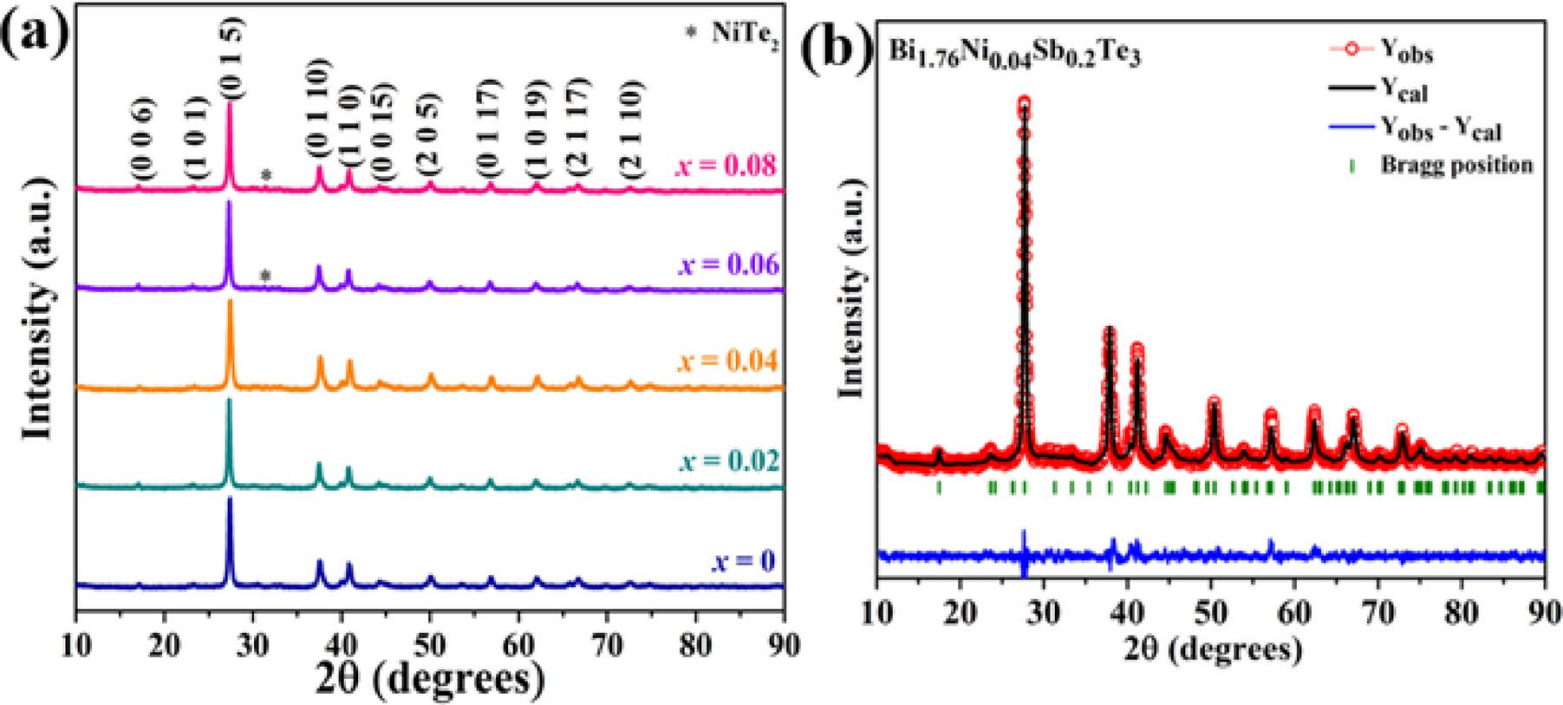
(**a**) XRD pattern and (**b**) Rietveld refinement plot of Bi_1.76_Ni_0.04_Sb_0.2_Te_3_ sample.

**Fig. 3:**
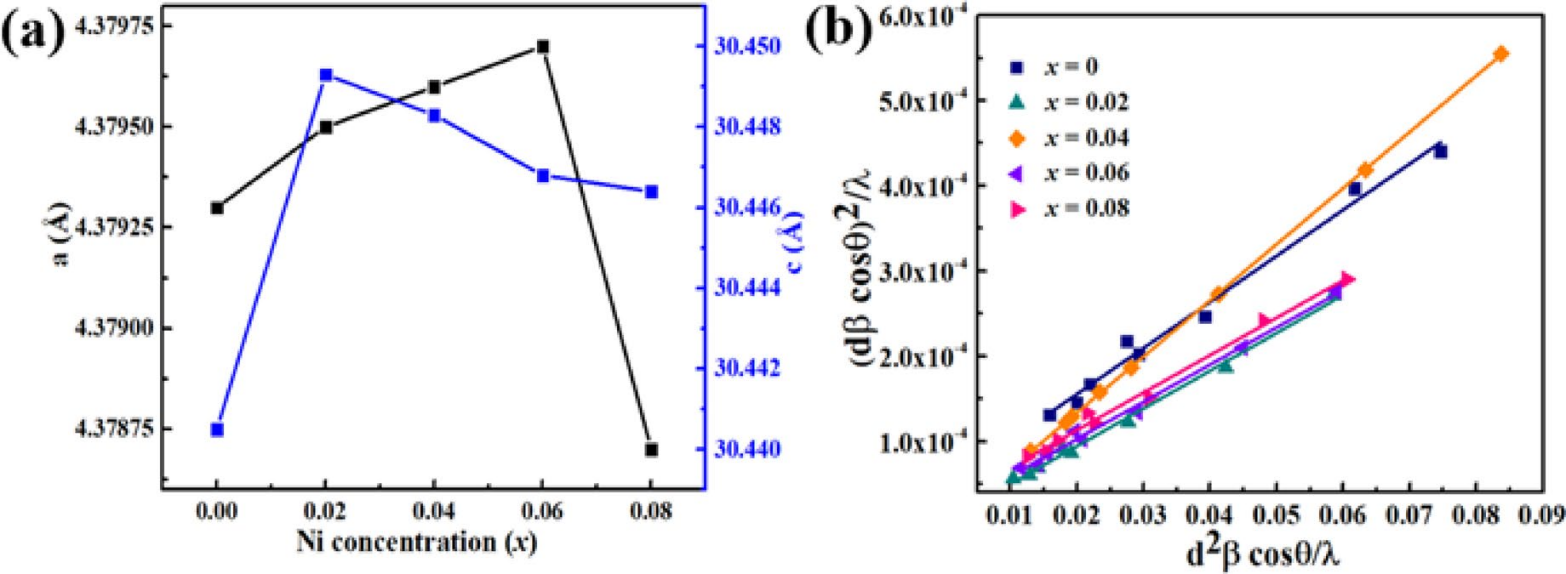
(**a**) Variation of lattice parameter with Ni concentration, (**b**) Size strain plot for the Bi_1.8−*x*_Ni_*x*_Sb_0.2_Te_3_ samples.

Powder X-ray diffraction pattern confirmed the phase homogeneity and crystallinity of prepared samples and the plots are depicted in Fig. 2a. Each diffraction peaks correspond to a rhombohedral lattice (space group ${\mathrm{R}}\overline{3}{\mathrm{m}}$) and matches with the JCPDS card No. #00-15-0863^25^. For higher Ni concentration (*x* = 0.06 and 0.08), a very less intense peak at ~ 31.4° is observed, suggesting the formation of NiTe_2_ secondary phase due to the solubility limit of Nickel. These observations are consistent with the previous reports^39,42^. Quantitative phase analysis was executed by Rietveld refinement for the observed experimental data using FULLPROF Suite and refinement plot for *x* = 0.04 sample is shown in the Fig. 2b^43^. Background contributions were modelled by linear interpolation, while the peak shapes were described by a pseudo-Voigt function. The refined line profiles well fitted with the measured data, resulting in goodness-of-fit values near 1.3. The procured refinement parameters, namely lattice constants, residual factors and *χ*^2^ are summarized in Table 1. As expected for low-level Ni substitution, the unit-cell dimensions remain essentially unchanged^44^. The variation of lattice parameters with Ni concentration is shown in Fig. 3a to verify the Vegard’s law behaviour of the samples, where lattice parameter has to vary linearly with the doping concentration^45^. The lattice parameter ‘a’ shows an approximately linear increase till *x* = 0.06 Ni concentration, indicating substitutional doping of Ni into the Bi site of the rhombohedral lattice. However, a deviation from linearity is observed at highest Ni concentration of *x* = 0.08. This behaviour may be attributed to the limited solubility of Ni and the appearance of a minor secondary phase as observed in XRD pattern. In contrast, the lattice parameter ‘c’ shows irregular variation with composition, which can be associated with the anisotropic layered structure of Bi–Sb–Te, where the interlayer spacing is highly sensitive to alloying, defects and strain. Such deviation from ideal Vegard’s law behaviour is commonly reported in Bi–Sb–Te based thermoelectric alloys^46,47^. The average crystallite size was estimated via the Scherrer and size-strain (SS) method and equations are given below.

1 $$D = \frac{k\lambda }{{\beta \cos \theta }}$$

2 $$\left( {\frac{{{\mathrm{d}}\beta \cos \theta }}{\lambda }} \right)^{2} = \frac{k}{D}\left( {\frac{{{\mathrm{d}}^{2} \beta \cos \theta }}{\lambda }} \right) + 4\varepsilon^{2}$$

where the shape factor is represented by *k* = 0.9, *D* denotes the crystallite size, *λ* = 1.5406 Å specifies the wavelength of Cu-kα radiation (X-ray source), *β* indicates the full width at half maximum and *ε* describes the lattice microstrain. The precision of the Scherrer method can be affected by factors such as microstrain. To address this issue, the microstrain has been quantified by implementing the SS plot which has depicted in Fig. 3b. However, the *D* calculated via the Scherrer equation corresponds closely within the error margin to the *D* determined by the SS plot. This is likely due to the low strain levels observed in the prepared samples. The observed reduction in microstrain after doping aligns with findings from existing studies^48,49^. The introduction of dual dopants (Sb and Ni) at the Bi site induced lattice dislocation $\left( \rho \right)$, which can be numerically calculated using the equation:

3 $$\rho = \frac{15\varepsilon }{{aD}}$$

where *a* represents the lattice parameter and *D* denotes the crystallite size^50^. The obtained values are listed in Table 2.

**Table 1:** Rietveld refined parameters and crystallite size of Bi_1.8−*x*_Ni_*x*_Sb_0.2_Te_3_ (0 ≤ *x* ≤ 0.08) samples.

Bi_1−*x*_Ni_*x*_Sb_0.2_Te_3_	*x* = 0	*x* = 0.02	*x* = 0.04	*x* = 0.06	*x* = 0.08
System	Rhombohedral	Rhombohedral	Rhombohedral	Rhombohedral	Rhombohedral
Space group	$R\overline{3}m$	$R\overline{3}m$	$R\overline{3}m$	$R\overline{3}m$	$R\overline{3}m$
*a* = *b* (Å)	4.3793(7)	4.3795(6)	4.3796(5)	4.3797(4)	4.3787(9)
*c* (Å)	30.4405(34)	30.4493(31)	30.4483(33)	30.4468(35)	30.4464(36)
*α* = *β* (°)	90	90	90	90	90
*γ* (°)	120	120	120	120	120”
Cell volume (Å^3^)	505.59(8)	505.96(7)	505.79(7)	505.78(4)	505.73(8)
R_p_	6.85	7.07	6.88	7.13	6.89
R_wp_	8.81	8.91	8.69	9.05	8.84
R_exp_	7.01	7.27	6.99	7.30	7.04
χ^2^	1.52	1.53	1.54	1.54	1.56
GOF	1.3	1.3	1.3	1.3	1.3
Crystallite size (Scherrer) in nm ± 5 nm	23	30	21	30	29
Crystallite size (SS plot) in nm ± 1 nm	25	31	21	32	31
Strain	0.0034	0.0011	0.0006	0.0019	0.0025
*ρ*(10^15^ m^−2^)	4.65	1.21	0.97	2.03	2.76

**Table 2:** Nominal and actual composition of the Bi_1.8-*x*_Ni_*x*_Sb_0.2_Te_3_ (0 ≤ *x* ≤ 0.08) samples.

Sample	Nominal composition	Actual composition
*x* = 0	Bi_1.8_Sb_0.2_Te_3_	Bi_1.93_Sb_0.17_Te_2.9_
*x* = 0.02	Bi_1.78_Ni_0.02_Sb_0.2_Te_3_	Bi_1.83_Ni_0.028_Sb_0.19_Te_2.92_
*x* = 0.04	Bi_1.76_Ni_0.04_Sb_0.2_Te_3_	Bi_1.84_Sb_0.043_Sb_0.198_Te_2.91_
*x* = 0.06	Bi_1.74_Ni_0.06_Sb_0.2_Te_3_	Bi_1.84_Ni_0.105_Sb_0.13_Te_2.91_
*x* = 0.08	Bi_1.72_Ni_0.08_Sb_0.2_Te_3_	Bi_1.78_Ni_0.118_Sb_0.169_Te_2.93_

### FESEM and EDX

**Fig. 4:**
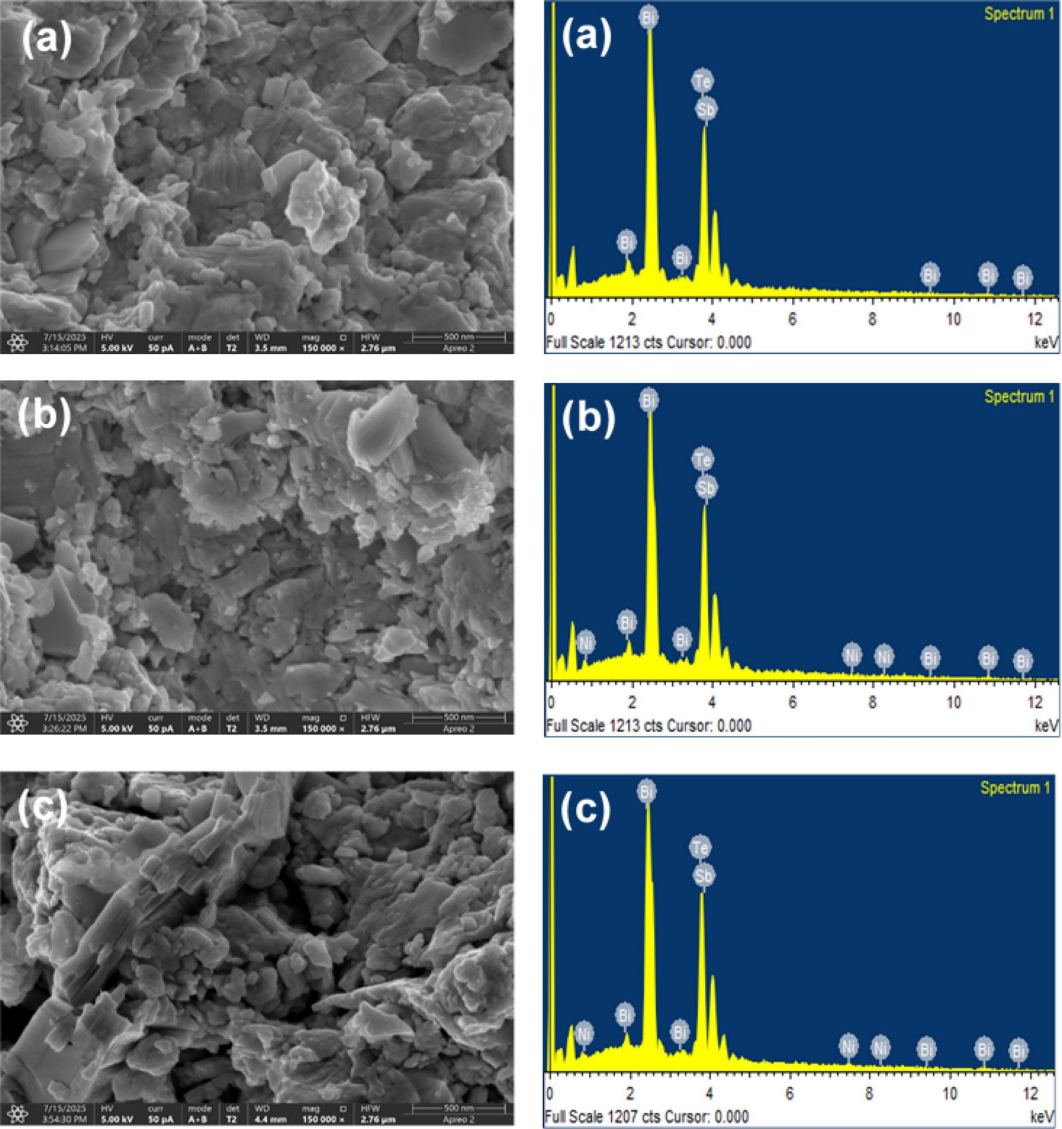
FESEM micrographs and EDX spectra of (**a**) Bi_1.8_Sb_0.2_Te_3_, (**b**) Bi_1.76_Ni_0.04_Sb_0.2_Te_3_, and (**c**) Bi_1.72_Ni_0.08_Sb_0.2_Te_3_ samples.

**Fig. 5:**
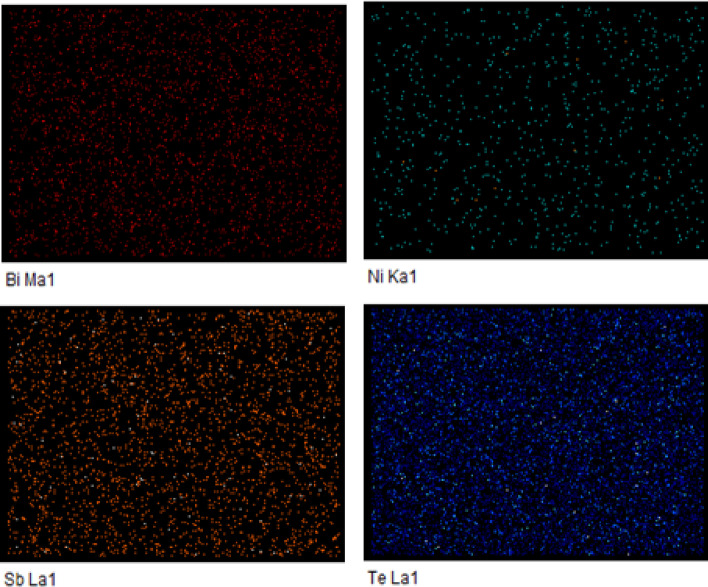
Elemental mapping of the Bi_1.76_Ni_0.04_Sb_0.2_Te_3_ samples.

FESEM images (Fig. 4) reveal a progressive evolution from well-ordered hexagonal plate-like crystallites in the pristine sample to increasingly fine, granular structures as the Ni content increases that indicates the successful incorporation of the dopant. The surface of the pristine sample displays smooth micrometre-scale flakes, which are characteristic of layered tetradymites. The evolution of granular morphology for higher Ni concentration is attributed to the combined effect of Ni alloying and the formation of the minor secondary phase which is observed in XRD. In layered Bi–Sb–Te alloys, the dopant incorporation introduces the lattice strain, defect formation and the compositional fluctuation which lead to the grain during the synthesis and sintering of the samples^51,52^. Moreover, the presence of a small amount of secondary phase can further hinder the microstructural growth of layered structure and favours the growth of the granular structure. The similar microstructural behaviour has been reported in Bismuth telluride based systems^48,53,54^. This systematic microstructural refinement from large, interconnected platelets to defect-rich granular aggregates formation reflects the changes in the TE properties. Energy dispersive X-ray spectroscopy (EDX) has quantified the presence of all elements within the samples. The nominal and actual compositions of the samples were determined using atomic percentage values obtained from EDX are presented in Table 2. The elemental mapping demonstrates a homogeneous distribution of elements throughout the samples are illustrated in Fig. 5.

### XPS

**Fig. 6:**
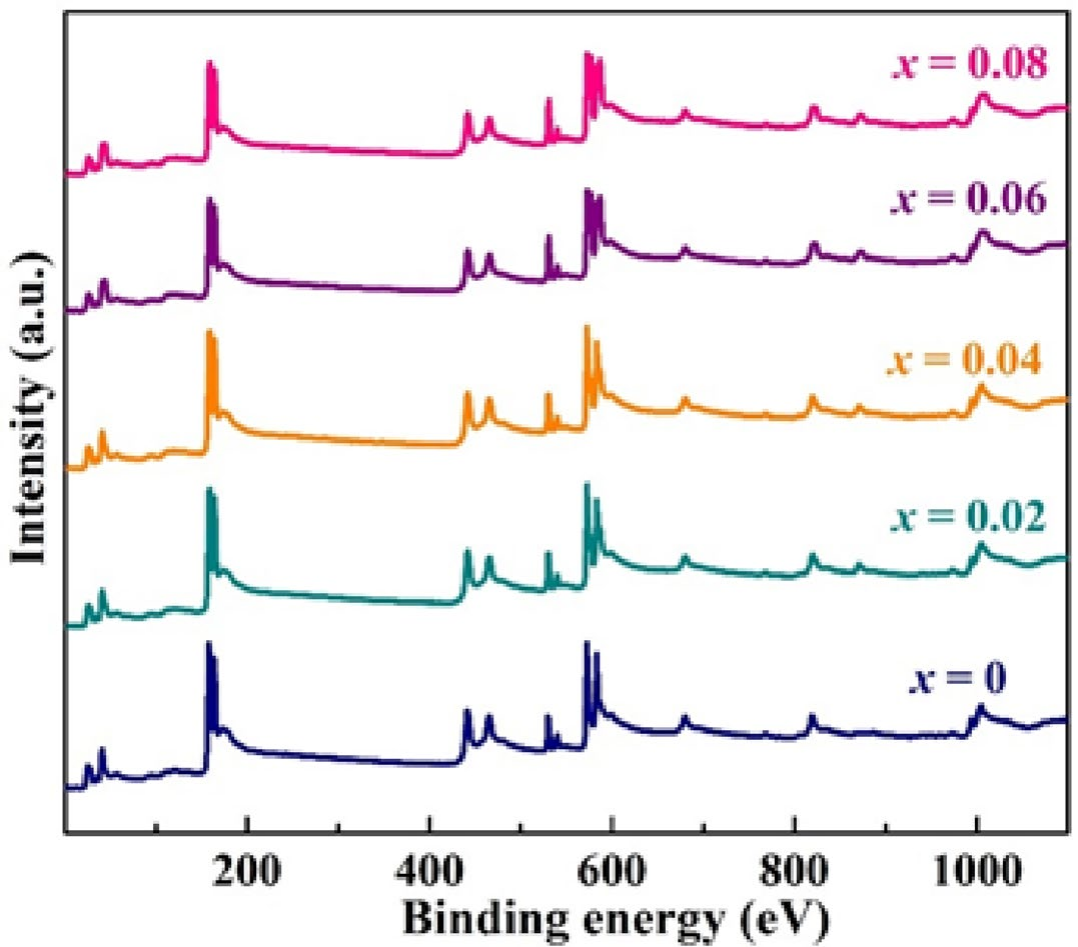
XPS survey spectra of the Bi_1.8−*x*_Ni_*x*_Sb_0.2_Te_3_ samples.

**Fig. 7:**
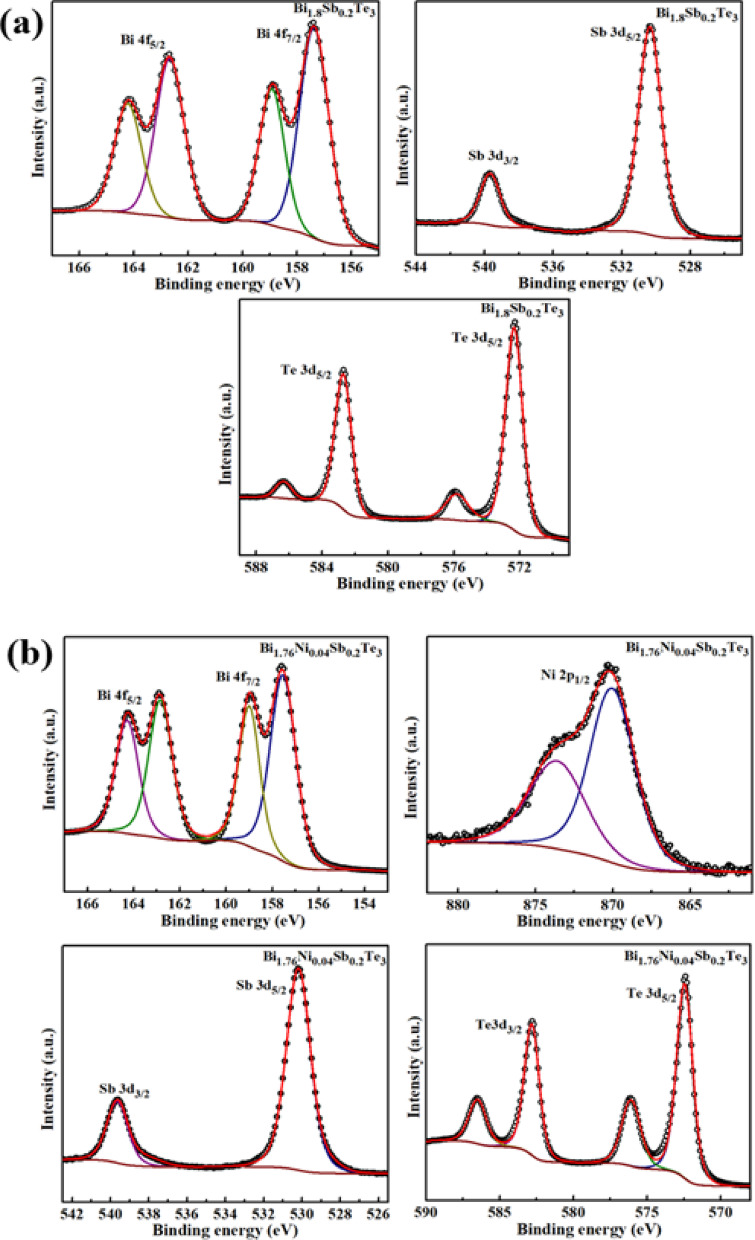
High-resolution X-ray photoelectron Spectra of (**a**) Pristine (Bi_1.8_Sb_0.2_Te_3_) and (**b**) Bi_1.76_Ni_0.04_Sb_0.2_Te_3_ sample.

Figure 6 illustrates the XPS survey spectra of all the prepared samples. Figure 7a, b shows the high resolution spectra of the pristine and Bi_1.76_Ni_0.04_Sb_0.2_Te_3_ samples respectively, presenting a detailed analysis of pristine and optimized composition that exhibits the highest TE performance. The high-resolution XPS spectra for the pristine and *x* = 0.04 sample confirm that Ni successfully substitutes for the Bi site within the Bi_1.8_Sb_0.2_Te_3_ matrix. The Bi 4*f*. region displays a spin–orbit doublet at 157.3 eV (4*f*_7/2_) and 162.6 eV (4*f*_5/2_) (ΔE = 5.3 eV) for pristine indicating that the Bi^3+^ oxidation state and 157.5 eV and 162.8 eV for *x* = 0.04 sample which has a 0.2 eV shift towards the higher binding energy upon Ni incorporation^55^. The Te 3*d* spectrum reveals binding energies of 572.3 eV (3*d*_5/2_) and 582.6 eV (3*d*_3/2_) (ΔE = 10.4 eV) of Te^2−^ in a layered structure, for the doped ~ 0.1 eV shift of binding energy to 572.4 eV and 582.7 eV. The Sb 3*d* peaks at 530 eV (3*d*_5/2_) and 539.4 eV (3*d*₃_/_₂) (9.4 eV), for doped sample at 530.1 eV and 539.5 eV confirms that Sb is in the + 3 oxidation state^56^. The core spectra for Ni doped sample of the Ni 2*p* spectrum reveals peaks at 870 eV and 873.6 eV which are corresponds to Ni^2+^ (2*p*_1/2_) state and higher Binding energy peak arise due to the multiplet splitting of Ni^2+^ state due to strong electron correlation effect, with no noticeable feature witnessed for the 2*p*_3/2_ level. This represents the strong matrix induced attenuation of the lower energy component due to covalent interactions between Ni and Te interactions and altered spin orbit coupling within the chalcogenide lattice^57^. Moreover, the positive systematic shift of Bi ~ 0.2 eV suggests a reduction in electron density of Bi atoms, which is consistent with replacement Bi by Ni atom. The small shift of ~ 0.1 eV of Sb and Te also evidenced the charge redistribution in the lattice which confirms the Ni is substitutionally incorporated in the Bi–Sb–Te lattice^50,55^. The XPS results demonstrate that incorporating Ni into the Bi sites maintains the intrinsic oxidation states of Bi, Sb and Te.

### Thermoelectric transport properties

**Fig. 8:**
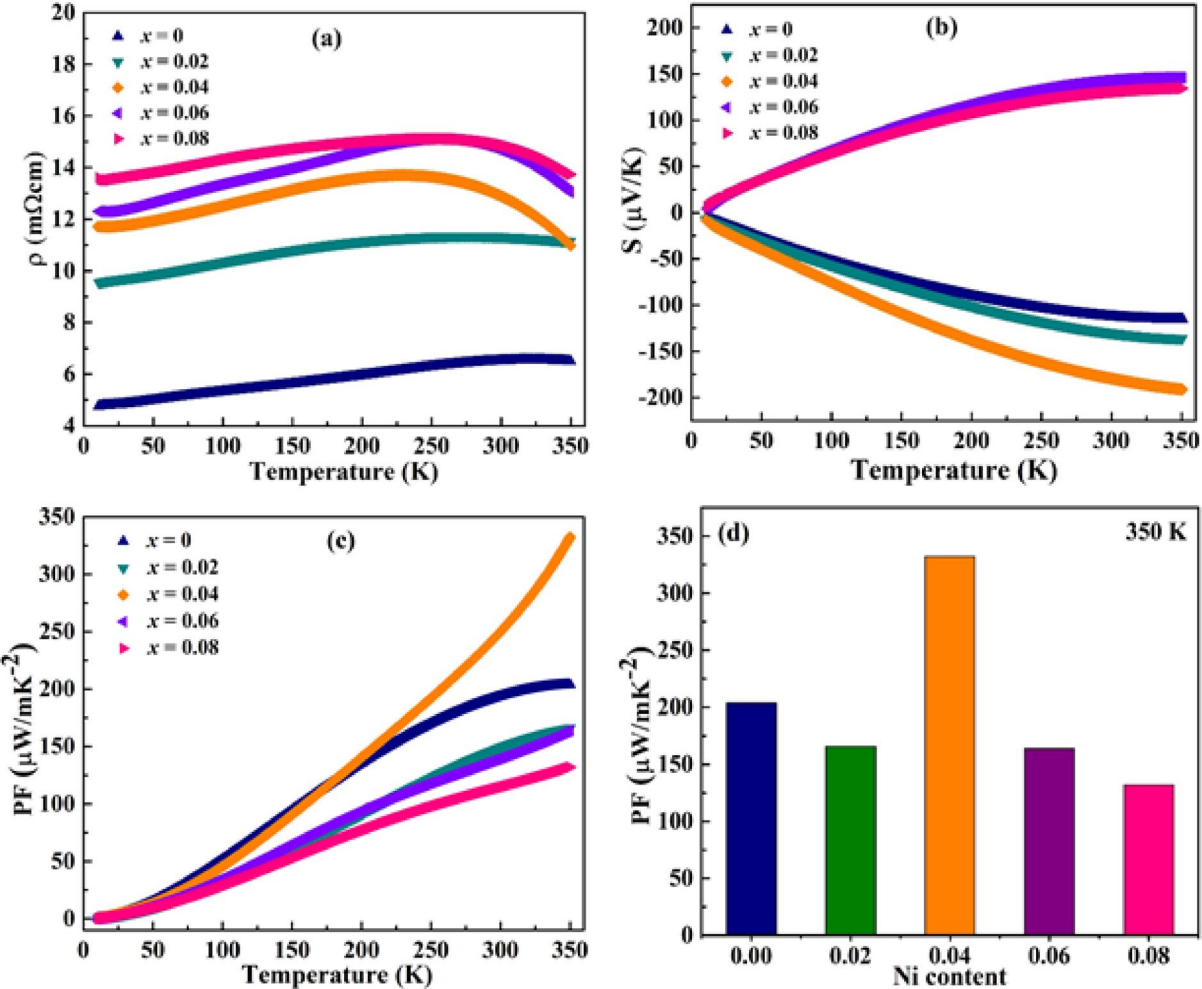
Temperature-dependent (**a**) electrical resistivity, (**b**) Seebeck coefficient, (**c**) power factor (*PF*), and (**d**) variation of *PF* with Ni concentration at 350 K of Bi_1.8−*x*_Ni_*x*_Sb_0.2_Te_3_ samples.

The temperature v/s resistivity *ρ*(T) profile of Bi_1.8-*x*_Ni_*x*_Sb_0.2_Te_3_ (Fig. 8a) shows that these samples behave as heavily doped degenerate semiconductors. As the temperature drops below ~ 100 K, the *ρ*(T) increases because the Fermi level becomes trapped in a narrow energy band. Therefore, the number of charge carriers remains nearly constant. Once scattering decreases the charged Ni impurities become the main scattering centers for electrical transport. Mixed conduction mechanism has been observed beyond 300 K, where resistivity curve begins to bend downwards denotes that minority electrons are thermally excited across the narrow gap of ~ 0.1 eV which is typical for Bi–Sb–Te alloys^32^. Doping with Ni raises the intrinsic excitation temperature by stabilizing the hole Fermi level deeper within the valence band. This process delays bipolar conduction and results in an overall increase in resistivity due to the doping^19^.

Room-temperature Hall effect data (Table 3) reveal that induced acceptor doping increases the hole concentration from 2.4 × 10^19^ cm^−3^ (*x* = 0) to 4.9 × 10^19^ cm^−3^ (*x* = 0.08). Yet it concurrently suppresses the carrier mobility from 15 to 9 cm^2^V^−1^ s^−1^ because each negatively charged $Ni_{Bi}^{\prime }$ defect intensifies Coulomb scattering and generates local strain fields. The resistivity depends on these parameters which is described by the equation

4 $$\rho = \frac{1}{{n{\mathrm{e}}\mu }} = \frac{{m^{*} }}{{n{\mathrm{e}}^{2} \left\langle \tau \right\rangle }}$$

where *n*, *μ*, and *τ* labelled as carrier concentration, mobility and relaxation time respectively. The resistivity continues to increase with Ni concentration with the maximum of 13.8 mΩcm at 350 K for *x* = 0.08 sample. Such increase is resulting from the reduction of carrier mobility, which has a more prominent effect than the enhanced charge carriers. These results are compatible with the SEM micrographs in which morphological change from interconnected layers to defect-rich granular aggregates can directly correlating with the observed rise in electrical resistivity. Theoretical calculations and experimental studies of these layered materials disclosed that the addition of transition metals like Ni leads to interaction between their *d* and the *p* electrons of Te^3,42,58^. This interaction creates new energy levels just above the main energy band resulting in more irregular energy bands. Hence it generates the further scattering sites that hinders the movement of charge carriers and also increased the number of available electronic states^42,58,59^. The dual effect of an increase in charge carriers and greater hindrance to their movement explains the complex behaviour observed in the samples. Mobility degradation arises from three distinct scattering mechanisms. Ionised impurity scattering dominates at low temperatures with temperature independent nature reflects the static coulombic potential with charged defects introduced by Ni doping. Then at mid temperature are marked by the neutral impurity and the alloy disorder scattering. These processes originate from the lattice distortions around Ni atoms. Lastly, acoustic phonon scattering controls transport above ~ 200 K. deformation potentials determine the strength of phonon scattering. The coupled behaviour of these three mechanisms produces a weak positive temperature coefficient of mobility that distinguishes the Ni doped materials from the pristine sample^60^. The combined trends in resistivity and the Hall effect highlight the delicate balance between carrier creation and mobility loss in tetradymites doped with transition metals. Moderate levels of Ni enhance the availability of holes but introduce significant impurity scattering that impedes the mobility.

**Table 3:** Charge carrier concentration and mobility derived from the Hall effect measurements, along with the estimated effective mass of carriers, energy band gap, measured density and porosity for Bi_1.8−*x*_Ni_*x*_Sb_0.2_Te_3_ samples.

Sample	*x* = 0	*x* = 0.02	*x* = 0.04	*x* = 0.06	*x* = 0.08
*n* (10^19^ cm^−3^)	2.45	3.67	4.07	4.57	4.89
*μ* (cm^2^/V-s)	15	14	12	10	9
m* (10^−31^ kg)	4.2	6.5	9.6	7.5	8.7
*E*_*g*_ (eV)	0.08	0.09	0.13	0.09	0.10
Density (g/cm^3^)	5.43	5.84	5.24	6.05	5.50
Porosity (%)	29.6	23.9	31.4	20.7	27.6

Figure 8b indicates that all Bi_1.8−*x*_Ni_*x*_Sb_0.2_Te_3_ samples presents an increasing Seebeck coefficient (*S*) with temperature which is the signature of degenerate semiconducting behaviour in which the Fermi level is positioned deep within a narrow band. According to the Mott relation^61^, the Seebeck coefficient is formulated as follows:

5 $$S = \frac{{8\pi^{2} k_{B}^{2} m^{*} T}}{{3{\mathrm{e}}h^{2} }}\left( {\frac{\pi }{3n}} \right)^{2/3}$$

where *S* scales inversely with the carrier concentration (*n)* and the sign of *S* indicates the dominant type of carrier and its magnitude is proportional to the effective mass ($m^{*}$). The lightly doped sample with *x* = 0.04 achieves the highest *S* of 191 µV/K at 350 K. It is due to the relatively low *n* of 4.07 × 10^19^ cm^−3^ and high $m^{*}$, which indicates a high density of states in the sample. The obtained Seebeck results are supported by the density measurement and porosity calculation of the prepared samples provided in the Table 3. The lowest density of 5.24 g/cm^3^ for sample *x* = 0.04 exhibits the highest porosity of 31% due to inverse behaviour. The pellets exhibit the visible porosity and low density, which is expected for the materials using cold pressing and conventional sintering method. The Seebeck coefficient exhibits highest for the high porous sample, which are associated with the enhanced carrier filtering and increased scattering due to pores and grain boundaries. Hence the density and porosity also validate the highest Seebeck coefficient behaviour for *x* = 0.04 sample. Both the pristine sample and the lightly Ni-doped samples (*x* ≤ 0.04) exhibit a negative Seebeck coefficient confirms that electron transport is n-type. The substitution of Ni^2+^ for Bi^3+^ introduces acceptor levels that partially reduce the number of electrons but do not change the majority carrier type.

Beyond *x* = 0.04 the density of ${\mathrm{Ni}}_{{{\mathrm{Bi}}}}^{\prime }$ acceptors exceed that of native electron donors (primarily ${\mathrm{V}}_{{{\mathrm{Te}}}}^{ \bullet \bullet }$) triggering the Fermi level to shift into the valence band. This results in a positive *S* for *x* = 0.06–0.08 where holes become the majority carriers. These results are further supported by the EDX composition analysis, which reveals the Te deficiency across all the samples. The deficiency indicates the presence of intrinsic Te vacancies that contribute to n-type conductivity. The introduction of Ni acceptor defects compensates for Te vacancy donors. At low Ni concentrations (*x* ≤ 0.04), there is partial compensation. While at higher concentrations (*x* ≥ 0.06), complete overcompensation leading to p-type conduction. This compensation effect not only reverses the sign of the Seebeck coefficient but also amplifies the intrinsic excitation temperature and that delays the formation of bipolarons by maintaining a large absolute value of Seebeck coefficient up to 350 K^20,38^.

In Kröger–Vink notation, these regimes can be described as below^9,62,63^:

6-i $${\mathrm{Te}}_{{{\mathrm{Te}}}} + {\mathrm{V}}_{{{\mathrm{Bi}}}}^{\prime \prime \prime } + 3{\mathrm{h}}^{ \bullet } \to {\mathrm{V}}_{{{\mathrm{Te}}}}^{ \bullet \bullet } + {\mathrm{Te}}_{{{\mathrm{Bi}}}}^{ \bullet } + 3{\mathrm{e}}^{\prime } \;({\mathrm{for}}\;{\mathrm{n-type}})$$

6-ii $${\mathrm{Ni}} + {\mathrm{Bi}}_{{{\mathrm{Bi}}}} + {\mathrm{V}}_{{{\mathrm{Te}}}}^{ \bullet \bullet } + 2{\mathrm{e}}^{\prime } \rightarrow {\mathrm{Ni}}_{{{\mathrm{Bi}}}}^{\prime } + {\mathrm{Bi}} + {\mathrm{Te}}_{{{\mathrm{Te}}}} + {\mathrm{h}}^{ \bullet } \;({\mathrm{for}}\;{\mathrm{p-type}})$$

The XPS results indicate the substitution of Ni at the Bi sites creates localized hybridized states of Ni *d* -Te *p* orbitals just above the valence band edge and XRD also indicates the formation of secondary phases of NiTe_2_ for the higher Ni concentration. These states act as deep acceptor levels that progressively shifts the Fermi level from the conduction band into the valence band and facilitating the transition from n-type to p-type conduction. Furthermore, the Ni–Te bonds distort the local lattice structure which enhances hole localization and stabilizes p-type conduction at higher concentrations of Ni^42^. The reduction in *S* in the p-type region is primarily attributed to an increase in carrier concentration. Table 3 further indicates that the $m^{*}$ increases from 4.2 × 10^−31^ kg (*x* = 0) to a maximum of 9.6 × 10^−31^ kg (*x* = 0.04) before decreasing slightly at higher *x* values, consistent with enhanced band-edge density of states due to Ni-induced resonant levels. The increase in effective mass suggests enhanced density of states near the Fermi level and due to the impurity induced band distortion and the carrier compensation. At higher Ni concentration $m^{*}$ decreases which is consistent with the transition of the carrier type from n-type to p-type. Moreover, effective mass depends on the band curvature responsible for the band in the carrier transport, the change in the carrier type and the compensation region where Fermi level close to the band edge enhances the density of states lead to the observed anomaly in the $m^{*}$ for *x* = 0.04 sample^64,65^. The narrow band gap (*Eg*) determined from high temperature *S*(*T*) fits using the equation *E*_*g*_ = 2*e*|*S*_max_|*T*_max_, ranges from 0.08 to 0.13 eV. The peaks observed in the samples with *x* = 0.04 indicate that moderate Ni doping widens the bandgap and increases $m^{*}$. This results in an enhanced *S* at room temperature, although it comes at the cost of reduced carrier mobility. These observations underscore the delicate balance between carrier concentration, effective mass and scattering effect. Figure 8c presents the temperature-dependent power factor (*PF*) for all Bi_1.8-*x*_Ni_*x*_Sb_0.2_Te_3_ samples. The power factor is calculated using the relation $PF = s^{2} /\rho$. The power factor increases with temperature for all compositions. The maximum value of 335 µW/mK^2^ at a Ni doping level of *x* = 0.04, as shown in Fig. 8d due to the maximum Seebeck coefficient for that sample.

**Fig. 9:**
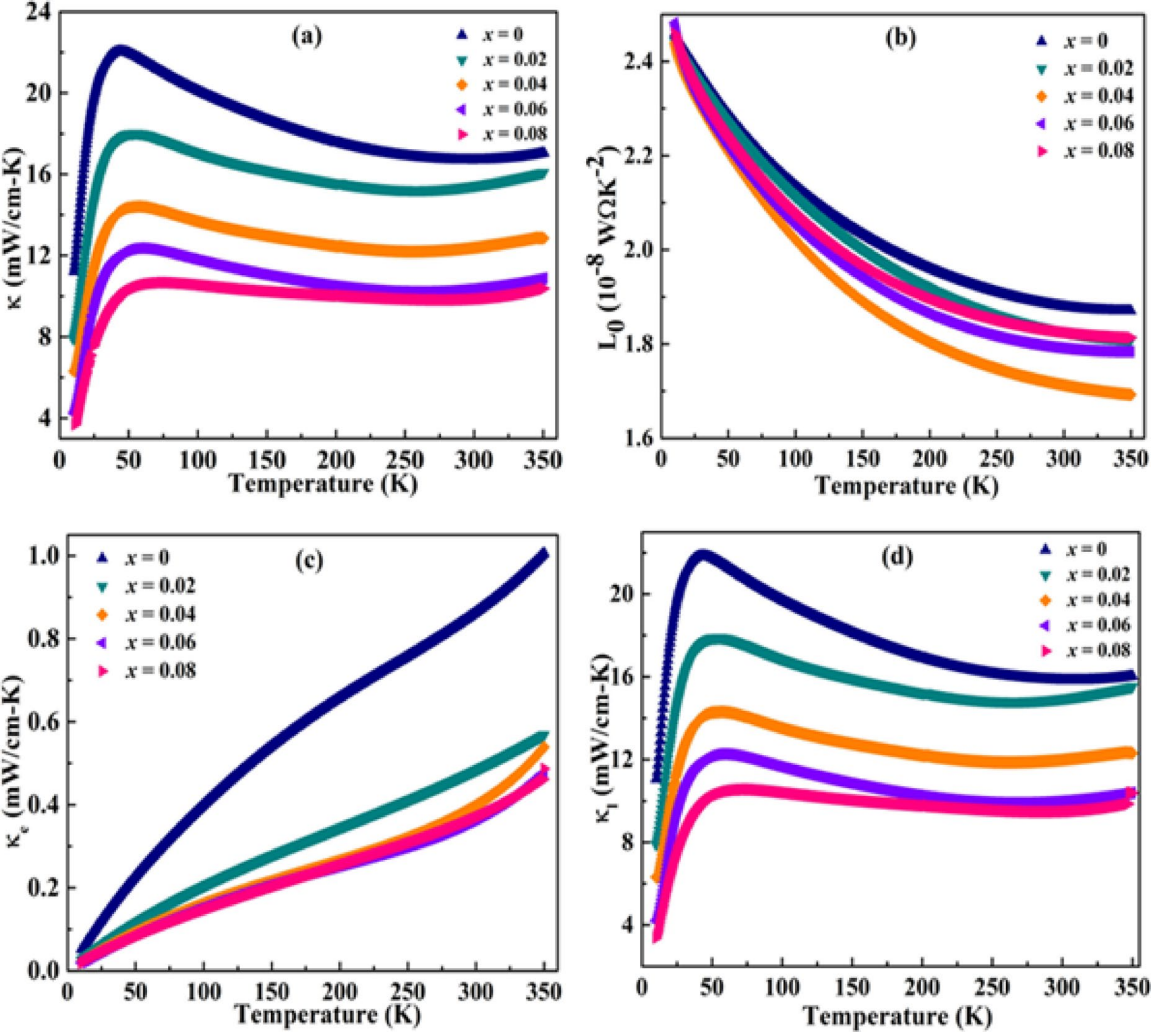
Temperature-dependent (**a**) total thermal conductivity, (**b**) Lorenz number, (**c**) electronic thermal conductivity, and (**d**) lattice thermal conductivity of Bi_1.8−*x*_Ni_*x*_Sb_0.2_Te_3_ samples.

The total thermal conductivity (κ) of Bi_1.8-*x*_Ni_*x*_Sb_0.2_Te_3_ (*x* = 0–0.08) samples is illustrated in Fig. 9a. All samples exhibit distinct peaks around 40–50 K because of the rapid increase in lattice specific heat and the long phonon mean free paths when boundary and point-defect scattering are minimal. κ systematically decreases due to the predominance of Umklapp scattering with a further rise in temperature to 350 K. The thermal conductivity decreases as the concentration of Ni doping increases. The lowest thermal conductivity value of about 10 mW/cmK at 350 K was obtained at a doping concentration of *x* = 0.08. The total thermal conductivity κ can be separated into its electronic ($\kappa_{e}$) and lattice ($\kappa_{l}$) components. The electronic part of thermal conductivity can be estimated via Wiedemann Franz law, which is given by:

7 $$\kappa_{e} = L_{0} T/\rho$$

where *ρ* is the electrical resistivity and $L_{0}$ is the Lorenz number. For heavily doped quasi-degenerate semiconducting materials, $L_{0}$ is calculated from measured Seebeck coefficient values using the approach given by Kim *et al*.rather than the traditional Sommerfeld value. This adjustment is necessary due to significant deviations caused by factors such as bipolar conduction, non-typical parabolic band structures and nickel-induced resonant states^66^. The equation for $L_{0}$ is given below

8 $$L_{0} = 1.5 + \exp \left( {\frac{ - \left| S \right|}{{116}}} \right) .$$

As shown in Fig. 9b, the decrease of the $L_{0}$ with temperature below the degenerate limit (2.44 × 10^–8^ WΩK^−2^) indicates a reduction in bipolar thermal conduction. This phenomenon is often attributed to strong carrier energy filtering or complex band structures that suppress electron–hole contributions to thermal transport. The electronic contribution $\kappa_{e}$ (Fig. 9c) systematically decreases as the Ni content increases. $\kappa_{e}$ for the pristine sample is approximately 1.0 mW cm^−1^ K^−1^ at 350 K, whereas for a Ni content of *x* = 0.08 $\kappa_{e}$ reduces below 0.6 mW cm^−1^ K^−1^. This trend mirrors the increase in *ρ* with Ni doping as ${\mathrm{Ni}}_{{{\mathrm{Bi}}}}^{\prime }$ acceptors not only introduce holes but also enhance carrier scattering leading to a reduction in electronic thermal conductivity.

By subtracting $\kappa_{e}$ from the total thermal conductivity, we derived the lattice thermal conductivity ($\kappa_{l}$). The temperature dependence of $\kappa_{l}$ reflects the influence of different phonon scattering mechanisms as depicted in Fig. 9d. The point defect and grain boundary scattering caused by Ni centres suppresses phonon–phonon interactions at the lowest temperatures. This leads to a notable rise in κ, characterized by a T^3^ relationship (κ α T^3^) up to 40 K. As the temperature rises beyond this range, three-phonon normal processes gradually become active and Umklapp processes start to contribute resulting in a steeper decline in conductivity from its maximum value. Between ~ 40 K and 100 K the combined effects of activated Umklapp scattering and defect scattering produce a clear T^−1^ relationship (κ α T^−1^) in thermal conductivity^67^. $\kappa_{l}$ decreases with increasing Ni concentration, falling from about 16 mWcm^−1^ K^−1^ at 350 K (*x* = 0) to around 9.8 mWcm^−1^ K^−1^ (*x* = 0.08).

The significant mass difference introduced by Ni substitution dramatically enhances phonons point-defect scattering in Bi_1.8_Sb_0.2_Te_3_. In the pristine sample, the 87 atomic mass unit (amu) difference between Bi and Sb already produces substantial disorder scattering. However, when Bi (209 amu) is replaced with Ni (58.7 amu) the mass difference increases to 150 amu. The observed mass difference strengthens the scattering effects contributes to the stronger point defect scattering on phonons which shortens their mean free paths. Nickel doping introduces Ni–Te bonds that are shorter and stronger than the native Bi-Te bonds, generating localized lattice distortions in the crystal. These structural perturbations supress heat carrying phonon propagation by reducing phonon relaxation time. The resulting strain fields modify the local energy landscape and significantly enhance phonon–phonon scattering processes thereby reducing the lattice thermal conductivity of the samples. The combined effects of changes in mass and strain fields lead to a reduction in the lattice thermal conductivity of Ni-doped samples^29,42^. This overall decrease in both $\kappa_{e}$ and $\kappa_{l}$ with Ni doping demonstrates the effectiveness of substitutional alloying in disrupting heat-carrying phonons and scattering charge carriers. Consequently, this minimizes parasitic thermal losses without significantly compromising electrical transport.

**Fig. 10:**
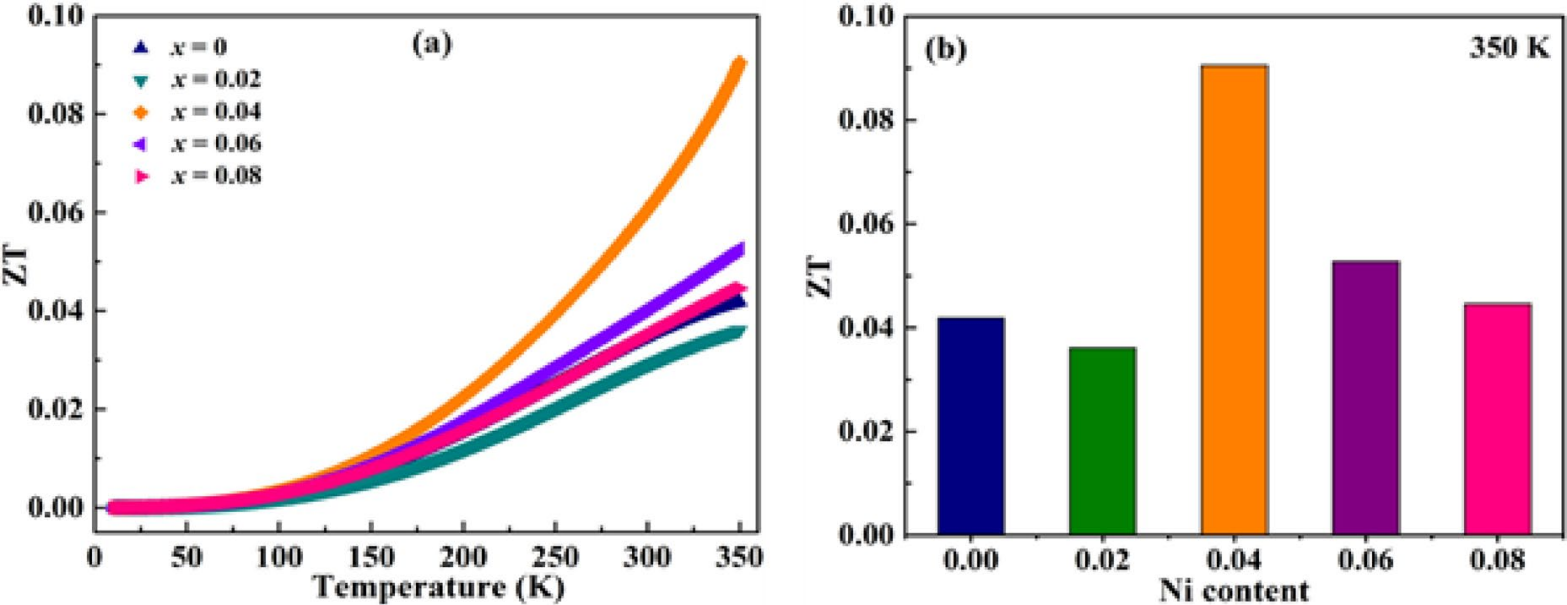
(**a**) Temperature-dependent figure of merit (*ZT*) and (**b**) the variation of *ZT* with Ni concentration at 350 K for Bi_1.8−*x*_Ni_*x*_Sb_0.2_Te_3_ samples.

The dimensionless thermoelectric figure of merit (*ZT*) was calculated using experimentally measured values of *ρ*, *S* and *κ*, expressed by the relation $ZT = S^{2} T/\rho \kappa$. Figure 10a represents the temperature dependence figure of merit of Bi_1.8-*x*_Ni_*x*_Sb_0.2_Te_3_ samples with 0 ≤ *x* ≤ 0.08. All compositions evidence an increase in *ZT* as the temperature rises. The pristine sample achieves a maximum *ZT* of 0.04 at 350 K. The variation in *ZT* values within the Ni-doped Bi_1.8-*x*_Ni_*x*_Sb_0.2_Te_3_ system is attributed to the effects of electronic and thermal transport caused by Ni substitution. *ZT* shows a slight decrease for the lowest doping (*x* = 0.02) sample. At higher Ni concentrations, ionized impurity scattering becomes the primary mobility limiting mechanism that supress the efficiency of the charge carrier transport. This enhanced scattering was reason for the reduction in the mobility effecting a twofold increase in the resistivity compared to the pristine. The maximum *ZT* of 0.09 is achieved at *x* = 0.04 as depicted in Fig. 10b. At this doping level, there is an optimal balance between the carrier concentration that achieved through the formation of ${\mathrm{Ni}}_{{{\mathrm{Bi}}}}^{\prime }$ acceptor defect and enhanced phonon scattering due to mass disorder and strain effects. This has enhanced *PF* and simultaneously reduced $\kappa_{l}$ thereby maximum ZT is observed for the sample with *x* = 0.04. In contrast to the previously reported Ni composite approach, where metallic interfacial scattering enhanced electron scattering and suppress the phonon scattering due to the formation of conductive metallic pathways leading to enhanced electrical resistivity and $\kappa_{l}$ resulting in reduction of TE efficiency. Hence substitutional doping enabling a more effective decoupling of charge and heat transport^41^. However, at higher doping concentrations (*x* ≥ 0.06) the increased defect scattering ultimately lowers carrier mobility, resulting in higher resistivity. The increased carrier concentration leads to a decrease in the Seebeck coefficient. As a result, *ZT* is reduced despite the observed decrease in thermal conductivity. Table 4 provides a comparison of the figure of merit for various cation-doped Bi–Sb–Te alloys as reported in the literature. Whereas, In the present work the investigation of transition metal (Ni) doped on n-type Bi–Sb–Te alloy provide a comprehensive analysis of the doping effect on structural and thermoelectric properties. The results illustrate that Ni inclusion induces notable modifications in carrier concentration, effective mass, carrier type transition, and lattice thermal conductivity, indicating strong influence on both the electronic band structure and phonon transport. This provides useful insight into the role of transition metal doping and fundamental understanding is important for designing improved thermoelectric materials through controlled band engineering and defect tuning. Therefore, the present work contributes to the understanding of doping-induced transport modification in Bi rich Bi–Sb–Te alloy based thermoelectric, which may be useful for future optimization of high-performance compositions.

**Table 4:** Comparison of *ZT* values for cation-doped Bi–Sb–Te alloys reported in the literature.

Sl No	Compound	Methodology	*ZT*	*T* (K)	References
1	Bi_0.5_Sb_1.45_Cu_0.05_Te_3_	Melting and cold pressing	0.74	442	^28^
2	Bi_0.5_Sb_1.46_Ni_0.04_Te_3_	Spark plasma sintering	1.38	433	^29^
3	Bi_0.5_Sb_1.4925_Mn_0.0075_Te_3_	Hot pressing method	1.3	430	^31^
4	Bi_0.5_Sb_1.44_Ti_0.06_Te_3_	Melting and hot pressing	1.1	323	^32^
5	Bi_0.5_Sb_1.4925_Yb_0.0075_Te_3_	Hot pressing method	1.3	330	^44^
6	Bi_1.76_Ni_0.04_Sb_0.2_Te_3_	Solid-state reaction	0.09	350	Present work

## Conclusion

This study elucidates the impact of nickel (Ni) substitution in modulating the TE transport parameters of Bi_1.8*-x*_Ni_*x*_Sb_0.2_Te_3_ alloys through defect engineering. The incorporation of Ni atoms at Bi sites induces structural, electronic and phononic modifications that enhance thermoelectric performance. Ni doping facilitates a refinement in the microstructure, resulting in a transition from ordered hexagonal platelets to disordered granular morphologies as confirmed by FESEM analyses. XPS confirms the chemical bonding in the Ni-doped Bi_1.8_Sb_0.2_Te_3_ alloy. Acceptor defects modulate carrier concentration inducing a transition from n-type to p-type with the Seebeck coefficient reaching a maximum of 191 μV/K at *x* = 0.04. The difference in atomic mass between Ni and Bi along with strain-field effects from shorter Ni–Te bonds creates phonon scattering mechanisms that reduce lattice thermal conductivity by as much as 40%. The optimization of transport parameters achieves a highest *ZT* of 0.09 at 350 K for *x* = 0.04 composition. This indicates that transition-metal substitutional doping can effectively decouple electronic and thermal transport in tetradymite thermoelectric materials. The study reveals the critical role of the defects in thermoelectric response and enables rational optimization of Bi–Sb–Te system via selective doping strategy.

## Data Availability

All data generated or analyzed during this study have been fully processed and are presented in the figures and tables within the manuscript. The raw datasets supporting the findings of this study are available from the corresponding author upon reasonable request.

## References

[1] Puthran, S., Hegde, G. S. & Prabhu, A. N. Review of chalcogenide-based materials for low-, mid-, and high-temperature thermoelectric applications. *J. Electron. Mater.* **53**, 5739–5768. 10.1007/s11664-024-11310-7 (2024).

[2] Sharma, S., Dwivedi, V. K. & Pandit, S. N. A review of thermoelectric devices for cooling applications. *Int. J. Green Energy.* **11**, 899–909. 10.1080/15435075.2013.829778 (2014).

[3] Rowe, D. M. (ed.) *CRC Handbook of Thermoelectrics* (CRC Press, 2018). 10.1201/9781420049718.

[4] Zheng, Y. et al. Defect engineering in thermoelectric materials: What have we learned?. *Chem. Soc. Rev.* **50**, 9022–9054. 10.1039/D1CS00347J (2021). 34137396 10.1039/d1cs00347j

[5] d’Angelo, M., Galassi, C. & Lecis, N. Thermoelectric materials and applications: A review. *Energies* **16**, 6409. 10.3390/en16176409 (2023).

[6] Wolf, M., Hinterding, R. & Feldhoff, A. High power factor vs. high zT—A review of thermoelectric materials for high-temperature application. *Entropy* **21**, 1058. 10.3390/e21111058 (2019).

[7] Soleimani, Z., Zoras, S., Ceranic, B., Shahzad, S. & Cui, Y. A review on recent developments of thermoelectric materials for room-temperature applications. *Sustain. Energy Technol. Assess.* **37**, 100604. 10.1016/j.seta.2019.100604 (2020).

[8] Sattar, M. & Yeo, W.-H. Recent advances in materials for wearable thermoelectric generators and biosensing devices. *Materials* **15**, 4315. 10.3390/ma15124315 (2022). 35744374 10.3390/ma15124315 PMC9230808

[9] Basit, A. et al. A review on high-performance flexible thermoelectrics: Material, device, and applications. *Microstructures*10.20517/microstructures.2024.56 (2025).

[10] Shi, X.-L., Li, N.-H., Li, M. & Chen, Z.-G. Toward efficient thermoelectric materials and devices: Advances, challenges, and opportunities. *Chem. Rev.* **125**, 7525–7724. 10.1021/acs.chemrev.5c00060 (2025). 40737123 10.1021/acs.chemrev.5c00060

[11] Romanenko, A. I., Chebanova, G. E., Chen, T., Su, W. & Wang, H. Review of the thermoelectric properties of layered oxides and chalcogenides. *J. Phys. D Appl. Phys.* **55**, 143001. 10.1088/1361-6463/ac3ce6 (2022).

[12] Zheng, Y. et al. Mechanically robust BiSbTe alloys with superior thermoelectric performance: A case study of stable hierarchical nanostructured thermoelectric materials. *Adv. Energy Mater.*10.1002/aenm.201401391 (2015).

[13] Zhang, J. et al. Enhanced thermoelectric performance of a quintuple layer of Bi_2_Te_3_. *J. Appl. Phys.*10.1063/1.4889921 (2014).25378712

[14] Yim, W. M. & Rosi, F. D. Compound tellurides and their alloys for Peltier cooling—A review. *Solid-State Electron.* **15**, 1121–1140. 10.1016/0038-1101(72)90172-4 (1972).

[15] Tang, Z., Hu, L., Zhu, T., Liu, X. & Zhao, X. High performance n-type bismuth telluride based alloys for mid-temperature power generation. *J. Mater. Chem. C.* **3**, 10597–10603. 10.1039/C5TC02263K (2015).

[16] Zhang, Q. et al. Evolution of the intrinsic point defects in Bismuth Telluride-based thermoelectric materials. *ACS Appl. Mater. Interfaces* **11**, 41424–41431. 10.1021/acsami.9b15198 (2019). 31612710 10.1021/acsami.9b15198

[17] Adekoya, A. H. & Snyder, G. J. Thermodynamic modeling of Bi_2_Te_3_ in the defect energy formalism. *Mater. Today Electron.* **9**, 100109. 10.1016/j.mtelec.2024.100109 (2024).

[18] Jang, J. et al. Development of p-type Bi_2−x_Sb_x_Te_3_ thermoelectric materials for power generation application exploiting synergetic effect of Sb alloying and repress process. *Appl. Surf. Sci.* **508**, 145236. 10.1016/j.apsusc.2019.145236 (2020).

[19] Goldsmid, H. Bismuth Telluride and its alloys as materials for thermoelectric generation. *Materials* **7**, 2577–2592. 10.3390/ma7042577 (2014). 28788584 10.3390/ma7042577 PMC5453363

[20] Zhou, Y. et al. n-Bi_2–x_Sb_x_Te_3_: A promising alternative to mainstream thermoelectric material n-Bi_2_Te_3–x_Se_x_ near room temperature. *ACS Appl. Mater. Interfaces* **12**, 31619–31627. 10.1021/acsami.0c07566 (2020). 32539321 10.1021/acsami.0c07566

[21] Hamawandi, B. et al. Facile solution synthesis, processing and characterization of n- and p-type binary and ternary Bi–Sb tellurides. *Appl. Sci.* **10**, 1178. 10.3390/app10031178 (2020).

[22] Zhao, Y., Dyck, J. S., Hernandez, B. M. & Burda, C. Enhancing thermoelectric performance of ternary nanocrystals through adjusting carrier concentration. *J. Am. Chem. Soc.* **132**, 4982–4983. 10.1021/ja100020m (2010). 20329792 10.1021/ja100020m

[23] Im, H.-J. et al. Control of carrier concentration in n-type hot-pressed Bi_1.8_Sb_0.2_Te_3_ alloys. *Jpn. J. Appl. Phys.* **43**, 3548. 10.1143/JJAP.43.3548 (2004).

[24] Im, H.-J., Kim, D.-H., Mitani, T. & Je, K.-C. Thermal properties for hot-pressed n-type (Bi_1−__*x*_Sb_*x*_)_2_Te_3_ alloys. *Jpn. J. Appl. Phys.* **43**, 1094. 10.1143/JJAP.43.1094 (2004).

[25] Ghose, P. K., Dalui, T. K., Chatterjee, A., Majumdar, S. & Giri, S. High magneto-Seebeck effect at room temperature in Bi_1.8_Sb_0.2_Te_3__−__y_Se_y_ crystal. *Appl. Phys. Lett.*10.1063/5.0053151 (2021).

[26] Thu Huong, N. et al. High thermoelectric performance at low temperature of p-Bi_1.8_Sb_0.2_Te_3.0_ grown by the gradient freeze method from Te-rich melt. *J. Alloys Compd.* **368**, 44–50. 10.1016/j.jallcom.2003.08.066 (2004).

[27] Hegde, G. S. & Prabhu, A. N. A review on doped/composite Bismuth chalcogenide compounds for thermoelectric device applications: Various synthesis techniques and challenges. *J. Electron. Mater.* **51**, 2014–2042. 10.1007/s11664-022-09513-x (2022).

[28] Cui, J. L. Thermoelectric performance of quaternary Cu–Bi–Sb–Te alloys prepared by cold pressing. *J. Alloys Compd.* **415**, 216–219. 10.1016/j.jallcom.2005.04.217 (2006).

[29] Bano, S., Misra, D. K., Tawale, J. S. & Auluck, S. Enhanced thermoelectric performance of Bi_0.5_Sb_1.5_Te_3_ via Ni-doping: A Shift of peak ZT at elevated temperature via suppressing intrinsic excitation. *J. Materiomics* **7**, 1264–1274. 10.1016/j.jmat.2021.03.003 (2021).

[30] Mun, H. et al. Fe-doping effect on thermoelectric properties of p-type Bi_0.48_Sb_1.52_Te_3_. *Materials* **8**, 959–965. 10.3390/ma8030959 (2015). 28787981 10.3390/ma8030959 PMC5455449

[31] Qin, H. et al. Improved thermoelectric performance of p-type Bi_0.5_Sb_1.5_Te_3_ through Mn doping at elevated temperature. *Mater. Today Phys.* **6**, 31–37. 10.1016/j.mtphys.2018.07.002 (2018).

[32] Wei, Z. et al. Enhanced room-temperature thermoelectric performance of p-type BiSbTe by reducing carrier concentration. *RSC Adv.* **9**, 2252–2257. 10.1039/C8RA09771B (2019). 35516121 10.1039/c8ra09771b PMC9059881

[33] Rana, N. et al. Optimizing the thermoelectric properties of transition metal doped Sb_2_Te_3_ mediated by carrier effective mass. *J. Appl. Phys.*10.1063/5.0239697 (2025).

[34] Mi, H.-X. et al. Effect of solder and barrier layer elements on the thermoelectric properties of Bi_0.5_Sb_1.5_Te_3_. *Mater. Res. Express* **6**, 106310. 10.1088/2053-1591/ab3cd5 (2019).

[35] P.K., J. S., Shah, M. & Pradyumnan, P. P. Magnetoelectric coupling and thermoelectric behaviors in Ni-doped CuCrO_2_ crystallites. *Chem. Eng. J.* **476**, 146568. 10.1016/j.cej.2023.146568 (2023).

[36] Ma, S. et al. High-pressure synthesis and excellent thermoelectric performance of Ni/BiTeSe magnetic nanocomposites. *J. Mater. Chem. A* **8**, 4816–4826. 10.1039/C9TA13284H (2020).

[37] Wei, Z. et al. Precise regulation of carrier concentration in thermoelectric BiSbTe alloys via magnetic doping. *ACS Appl. Mater. Interfaces* **12**, 20653–20663. 10.1021/acsami.0c02408 (2020). 32286043 10.1021/acsami.0c02408

[38] Lee, N. et al. The electrical conduction-type transition in Se-free Bi_2_Te_3_ thermoelectric materials by decoration of Ni nanoparticles. *ACS Appl. Energy Mater.* **7**, 8807–8813. 10.1021/acsaem.4c01745 (2024).

[39] Hu, X. et al. The effect of Ni/Sn doping on the thermoelectric properties of BiSbTe polycrystalline bulks. *J. Solid State Chem.* **277**, 175–181. 10.1016/j.jssc.2019.06.006 (2019).

[40] Zhang, J. et al. Enhanced contact performance and thermal tolerance of Ni/Bi_2_Te_3_ joints for Bi_2_Te_3_-based thermoelectric devices. *ACS Appl. Mater. Interfaces* **15**, 22705–22713. 10.1021/acsami.3c01904 (2023). 37126364 10.1021/acsami.3c01904

[41] G., P. et al. Investigations on the impact of nickel doping on thermoelectric properties in n-type Bi_1.8_Sb_0.2_Te_3_ alloy. *RSC Adv.* **15**, 34211–34224. 10.1039/D5RA05504K (2025). 40979952 10.1039/d5ra05504k PMC12444876

[42] Bano, S. et al. Enhanced thermoelectric performance of Ni_*x*_Bi_0.5_Sb_1.5_Te_3_*via in situ* formation of NiTe_2_ channels. *ACS Appl. Energy Mater.* **5**, 14127–14135. 10.1021/acsaem.2c02675 (2022).

[43] Rodríguez-Carvajal, J. Recent advances in magnetic structure determination by neutron powder diffraction. *Physica B Condens. Matter* **192**, 55–69. 10.1016/0921-4526(93)90108-I (1993).

[44] Qin, H. et al. Rare earth ytterbium enhanced thermoelectric properties of p-type Bi_0.5_Sb_1.5_Te_3_. *Appl. Phys. Lett.*10.1063/1.5091577 (2019).

[45] Denton, A. R. & Ashcroft, N. W. Vegard’s law. *Phys. Rev. A (Coll. Park).* **43**, 3161–3164. 10.1103/PhysRevA.43.3161 (1991). 10.1103/physreva.43.3161 9905387

[46] Malik, K. et al. Sb concentration dependent structural and resistive properties of polycrystalline Bi-Sb alloys. *J. Appl. Phys.*10.1063/1.4759137 (2012).

[47] Femi, O. E., Ravishankar, N. & Chattopadhyay, K. Microstructure evolution and thermoelectric properties of Te-poor and Te-rich (Bi,Sb)_2_Te_3_ prepared via solidification. *J. Mater. Sci.* **51**, 7254–7265. 10.1007/s10853-016-0008-3 (2016).

[48] Abishek, N. S. & Gopalakrishna Naik, K. Influence of gallium doping on structural and thermoelectric properties of bismuth telluride. *J. Cryst. Growth* **565**, 126141. 10.1016/j.jcrysgro.2021.126141 (2021).

[49] Rani, K., Gupta, V., Ranjeet, & Pandey, A. Improved thermoelectric performance of Se-doped n-type nanostructured Bi_2_Te_3_. *J. Mater. Sci. Mater. Electron.* **34**, 1074. 10.1007/s10854-023-10490-y (2023).

[50] Musah, J. et al. Ultralow thermal conductivity in dual‐doped n‐type Bi_2_Te_3_ material for enhanced thermoelectric properties. *Adv. Electron. Mater.*10.1002/aelm.202000910 (2021).

[51] Zhu, B. et al. Cold-sintered Bi_2_Te_3_-based materials for engineering nanograined thermoelectrics. *ACS Appl. Energy Mater.* **5**, 2002–2010. 10.1021/acsaem.1c03540 (2022).

[52] Guo, X. et al. Effect of doping indium into a Bi_2_Te_3_ matrix on the microstructure and thermoelectric transport properties. *RSC Adv.* **6**, 60736–60740. 10.1039/C6RA09767G (2016).

[53] Kaleemullah, N. S., Malaidurai, M., Thangavel, R. & Kumar, J. Investigation on the structural and magnetic properties of M_x_Bi_2–x_Te_3_ (M = Gd, Fe, Cr) (x = 0, 1) using colloidal hot-injection method. *Bull. Mater. Sci.* **45**, 53. 10.1007/s12034-021-02632-x (2022).

[54] Thakur, V., Upadhyay, K., Kaur, R., Goyal, N. & Gautam, S. Investigating phase transition and morphology of Bi-Te thermoelectric system. *Mater. Today Adv.* **8**, 100082. 10.1016/j.mtadv.2020.100082 (2020).

[55] Arévalo-López, E. P. et al. Effect of Fe on Bi_2_Te_3_: Structure, magnetic properties, and XPS valence band. *J. Alloys Compd.* **899**, 163297. 10.1016/j.jallcom.2021.163297 (2022).

[56] Zhu, J. et al. Permeable carbon fiber based thermoelectric film with exceptional EMI shielding performance and sensor capabilities. *J. Adv. Ceram.* **13**, 1119–1131. 10.26599/JAC.2024.9220922 (2024).

[57] Grosvenor, A. P., Biesinger, M. C., Smart, R. S. C. & McIntyre, N. S. New interpretations of XPS spectra of nickel metal and oxides. *Surf. Sci.* **600**, 1771–1779. 10.1016/j.susc.2006.01.041 (2006).

[58] Jena, A., Lee, S.-C. & Bhattacharjee, S. Tuning the lattice thermal conductivity in bismuth telluride via Cr alloying. *Phys. Rev. Appl.* **15**, 064023. 10.1103/PhysRevApplied.15.064023 (2021).

[59] Gao, Q. et al. Orbital textures and evolution of correlated insulating state in monolayer 1T phase transition metal dichalcogenides. *Nat. Commun.* **16**, 3784. 10.1038/s41467-025-59228-w (2025). 40263335 10.1038/s41467-025-59228-w PMC12015590

[60] Kim, J. H., Back, S. Y., Yun, J. H., Lee, H. S. & Rhyee, J.-S. Scattering mechanisms and suppression of bipolar diffusion effect in Bi_2_Te_2.85_Se_0.15_I_x_ compounds. *Materials* **14**, 1564. 10.3390/ma14061564 (2021). 33810161 10.3390/ma14061564 PMC8004870

[61] Huang, Z. et al. Improving the thermoelectric performance of p-type PbSe *via* synergistically enhancing the Seebeck coefficient and reducing electronic thermal conductivity. *J. Mater. Chem. A* **8**, 4931–4937. 10.1039/C9TA13927C (2020).

[62] Kröger, F. A. & Vink, H. J. Relations between the concentrations of imperfections in crystalline solids. 307–435. 10.1016/S0081-1947(08)60135-6 (1956).

[63] Oh, M. W. et al. Antisite defects in *n* -type Bi_2_(Te,Se)_3_: Experimental and theoretical studies. *J. Appl. Phys.*10.1063/1.4870818 (2014).

[64] Pei, Y. et al. Convergence of electronic bands for high performance bulk thermoelectrics. *Nature* **473**, 66–69. 10.1038/nature09996 (2011). 21544143 10.1038/nature09996

[65] Snyder, G. J. & Toberer, E. S. Complex thermoelectric materials. *Nat. Mater.* **7**, 105–114. 10.1038/nmat2090 (2008). 18219332 10.1038/nmat2090

[66] Kim, H.-S., Gibbs, Z. M., Tang, Y., Wang, H. & Snyder, G. J. Characterization of Lorenz number with Seebeck coefficient measurement. *APL Mater.*10.1063/1.4908244 (2015).

[67] Yao, M., Opeil, C., Wilson, S. & Zebarjadi, M. Experimental determination of phonon thermal conductivity and Lorenz ratio of single-crystal bismuth telluride. *MRS Commun.* **7**, 922–927. 10.1557/mrc.2017.118 (2017).

